# Optimization of inoculum production of *Stemphylium botryosum* for large-scale resistance screening of lentils

**DOI:** 10.1186/s13007-024-01177-4

**Published:** 2024-04-04

**Authors:** Mario González, Eleonora Barilli, Nicolas Rispail, Diego Rubiales

**Affiliations:** Institute for Sustainable Agriculture, CSIC, Avda. Menéndez Pidal s/n, Cordoba, 14004 Spain

**Keywords:** Large-scale screening, *Lens culinaris*, Resistance evaluation, Stemphylium blight

## Abstract

**Background:**

Stemphylium blight incited by *Stemphylium botryosum* poses a significant threat to lentil crops worldwide, inducing severe defoliation and causing substantial yield losses in susceptible varieties under favorable conditions. While some moderate levels of resistance have been identified within lentil germplasm, a low number of resistant cultivars are available to farmers. Adding to the common constraints of resistance breeding, a notable challenge is generating a sufficient number of spores for large-scale screenings, which are essential for pinpointing additional sources of resistance for integration into breeding programs. Therefore, there is a pressing need to improve existing screening methods and tailor them for large-scale material selection. In pursuit of this objective, a protocol for the efficient production of fungal material has been adapted.

**Results:**

Optimization of fungal material production was successfully achieved by comparing the use of fungal mycelia and spores. Spore production was found to be optimal when produced on solid V8-PDA(hi) medium, while liquid Richard’s medium was identified as superior for mycelium yield. Furthermore, a refined screening method was developed by evaluating the resistance of six lentil accessions to stemphylium blight. This assessment included the use of either fungal mycelia (at densities ranging from 1 to 5 g L^− 1^) or spores (with densities ranging from 5 × 10^4^ to 2 × 10^5^ conidia mL^− 1^) under three different relative humidity levels (from 50 to 100%). Both humidity levels and inoculum dose significantly influenced the final disease rating (DR) and the relative Area Under the Disease Progress Curve (rAUDPC). Differences among genotypes in final symptom severity (DR) became more pronounced after inoculation with inoculum densities of 5 g L^− 1^ of mycelium or of 10^5^ and 2 × 10^5^ conidia mL^− 1^ of spore under 100% relative humidity. Given the challenges associated with the large-scale production of *S. botryosum* spores, inoculations with 5 g L^− 1^ of mycelium is highly recommended as a practical alternative for conducting mass-scale screenings.

**Conclusions:**

The findings from this study underscore the critical importance of maintaining high level of humidity during inoculation and disease progression development for accurately assessing resistance to stemphylium blight. The optimization of mycelial production for suspension inoculation emerges as a more reliable and efficient approach for conducting large-scale screening to assess germplasm resistance against stemphylium blight in lentil crops.

**Supplementary Information:**

The online version contains supplementary material available at 10.1186/s13007-024-01177-4.

## Background

Lentil (*Lens culinaris* ssp. *culinaris* Medik.) is a cool-season food legume widely cultivated worldwide. Its consumption has surged significantly in recent years, outpacing the growth of other legumes [1]. This nutritious pulse is an important source of proteins, carbohydrates, fibers, vitamins and micronutrients in the human diet [2, 3]. Nonetheless, the global average grain yield of lentil remains low, primarily due to its susceptibility to a number of stresses [4]. Among these, stemphylium blight, caused by the Ascomycetes *Stemphylium botryosum* Wallr., is one of the most damaging foliar diseases seriously impacting lentil production in many countries where lentils are grown [5]. Stemphylium blight was first reported in Bangladesh in 1986 [6]. It was initially considered as a disease with limited impact, affecting lentil crops only locally. However, the pathogen has steadily increased in virulence and is currently responsible for up to 80% lentil yield losses [7]. Furthermore, over recent decades, its incidence has also increased in different regions of the world, notably in Canada [8], India, Nepal and North America [3, 9].

Stemphylium blight typically manifests in the field during the flowering or early pod formation stages, especially in temperate and humid environmental conditions, leading to prolonged leaf wetness [3]. The characteristic symptoms of this disease become apparent on the leaflets as small, pin-headed light brown to tan-colored spots, rapidly covering the leaf surface within 2 to 3 days, ultimately causing swift leaf drop and stem twisting [10, 11]. Infected stems tend to bend down and dry up. Flowers and pedicels can also be infected, leading to flower abortion [12]. These symptoms are particularly pronounced on the upper canopy; however, under optimal conditions, the entire plant can be affected, resulting in biomass loss and reduced seed yield [12].

The primary infection and propagation of the disease are attributed to conidia and ascospores, which originate from plant debris [13–15]. Furthermore, infected seeds facilitate pathogen’s dissemination across regions and serve as an initial source of inoculum during the season [16]. Secondary infections are initiated by airborne conidia, which develop across multiple generations on specific conidiophores present on the leaf surface [12] and can be spread on windy days over large areas under favorable environmental conditions [12, 17]. The latter, together with the lack of resistant varieties, has the potential to trigger acute epidemics and severe yield losses in endemic areas [3].

Managing stemphylium blight disease presents significant challenges due to its ability to rapidly generate substantial amounts of secondary inoculum under favorable environmental conditions. For *S. botryosum*, temperature and moisture serve as primary environmental factors influencing conidial germination and disease progression. In fact, the optimal conditions leading to high disease incidence involve an average daytime temperature of 18–20 °C, morning relative humidity (RH) exceeding 85%, rainfall, and the presence of a thin layer of moisture on the leaf surface for over 48 h [7, 12, 18]. Integrated disease management strategies for stemphylium blight in lentil include adjusting sowing time, implementing crop rotation with nonhost plants, practicing field sanitation, treating seeds by physical and chemical methods, deploying effective biocontrol agents, and strategically applying fungicides such as tebuconazole, chlorothalonil, procymidone, mancozeb and iprodione [19].

In this scenario, the search for resistance to *S. botryosum* stands as a critical imperative. However, progress in resistance breeding has been slow thus far. Several studies have explored the identification of sources of resistance to *S. botryosum* across different *Lens* species. While lentil cultivars offer limited resistance [3], some degree of partial resistance is available in landraces and wild relatives that are crossable with cultivated lentil [19–21].

In a 2007 genetic analysis [20], it was posited that resistance to stemphylium blight in the lentil hybrid Barimasur-4 × CDC Milestone was inherited in a quantitative manner. Expanding on this, in 2010 the presence of between one and three quantitative trait loci (QTLs) was reported in a recombinant inbred line (RIL) cross of ILL6002 and ILL5888, observed over two successive cropping seasons, with a consistent QTL identified in both years [11]. Furthermore, they pinpointed a marker closely linked to this QTL, suggesting its potential application in marker-assisted breeding programs. Nevertheless, understanding the genetic backgrounds of other lentil species is crucial to enhance our knowledge of lentil genetic resistance against *S. botryosum*. For this purpose, artificial inoculation with conidial suspensions obtained through fungal growth on culture media is a common practice to select germplasm under controlled and semi controlled conditions [8, 12, 22]. However, *S. botryosum* has a limited sporulation ability in axenic culture [11, 13], hampering the large-scale spore production needed to screen large germplasm collections or large segregating populations. The optimization of inoculum production and of the inoculation method as a whole is therefore urgently needed to allow large-scale screening of lentil germplasm and populations. Recent studies have resorted to mycelial suspensions for artificial inoculations [9]. However, a comprehensive understanding of the impact of inoculum type, inoculum concentration, growth conditions and their interaction with genotype has yet to be determined.

Hence, there is a pressing need to develop precise screening techniques for large-scale germplasm evaluation of lentil resistance to stemphylium blight to unearth new sources of resistance and to validate the potential of breeding materials under development. The primary objectives of this research were to optimize inoculum production (conidia and mycelium), estimate their infectivity and investigate the influence of air humidity on disease development in order to develop an efficient and robust large-scale screening method for stemphylium blight resistance screening in lentil.

## Results

### Influence of culture medium selection on spore and mycelium production

**Fig. 1:**
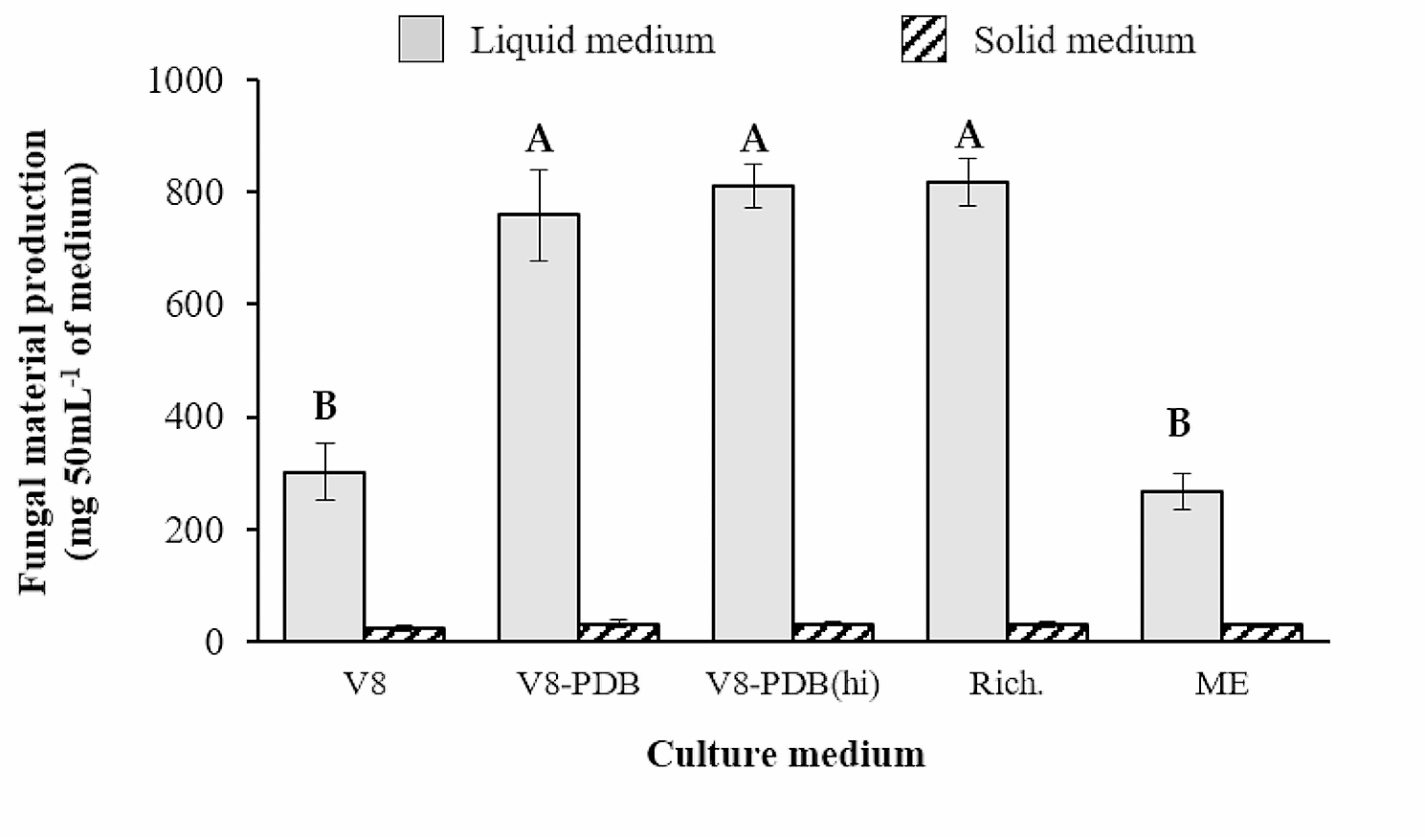
Production of fungal material. The graphic shows the mean weight of fungal material produced in liquid and solid media after 20 days of growth. Vertical bars are standard errors for *n* = 5. Different letters indicate significant differences in liquid media based on the LSD test (α ≤ 0.05)

Both solid and liquid culture media exhibited marked variations in terms of the weight of fungal material produced (Fig. 1). The highest production was achieved in liquid media in comparison with solid media (*p* < 0.0001), ranging from 266.8 mg in Malt extract (ME) to 817.2 mg per 50 mL of Richard medium (Rich.). In contrast, no significant differences were observed between the fungal biomass collected from solid media, which ranged from 23.7 mg (V8-A) to 31.1 mg (V8-PDA).

**Fig. 2:**
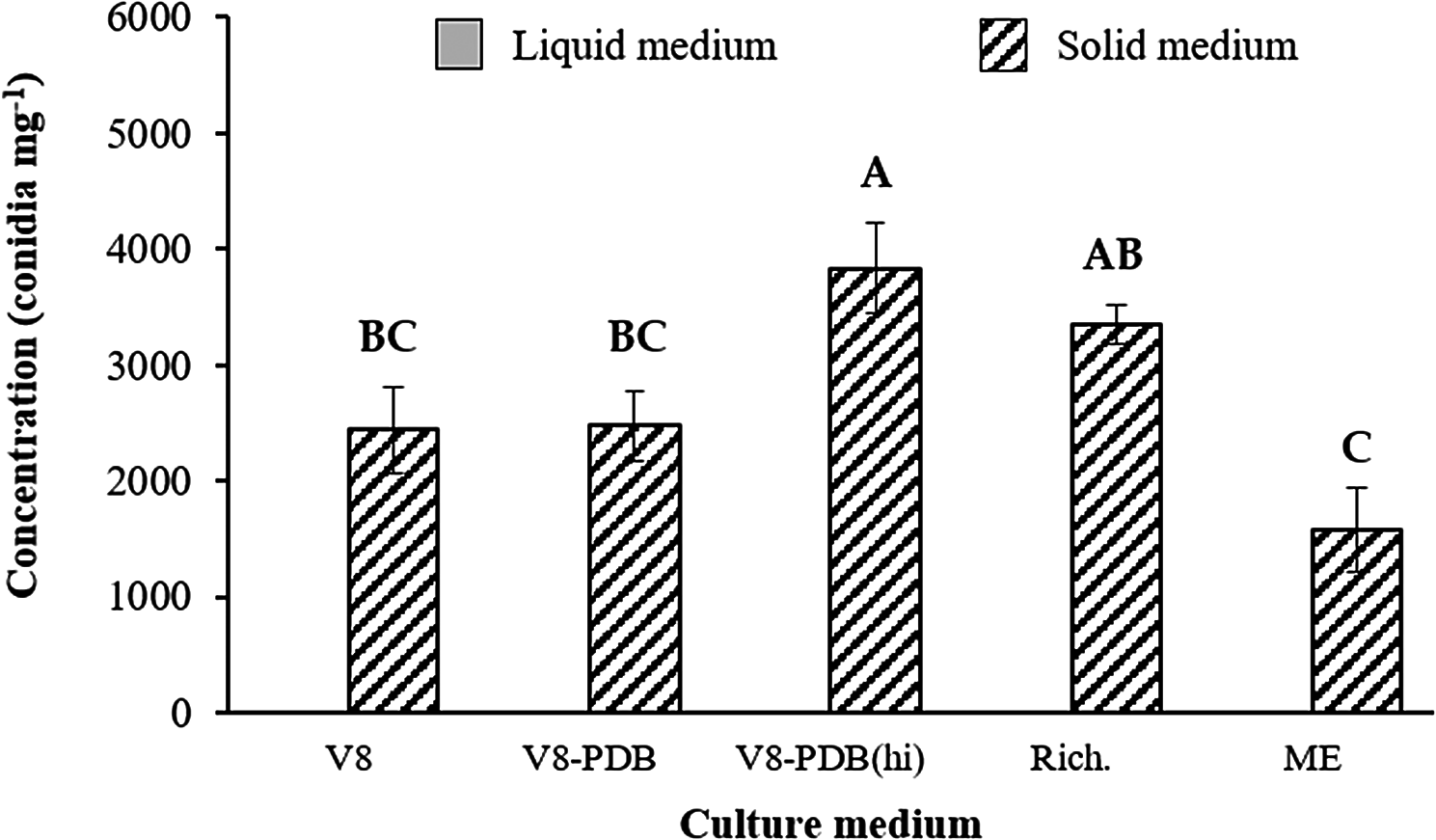
Comparison of the amount of *Stemphylium botryosum* conidia produced by the different axenic media. The graph presents the conidia densities produced in solid culture media after 20 days of incubation. No conidia were produced in liquid media. Data presented are the mean values ± standard errors (vertical bars) for *n* = 5. Different letters indicate significant differences on solid media based on the LSD test (α ≤ 0.05)

While the fungal biomass produced by the solid media was similar, large variation was detected between media in terms of conidia formation (*p* = 0.001) (Fig. 2). Conidia were never produced in liquid medium. The maximum production of conidia was achieved on V8-PDA(hi), averaging 3.8 × 10^3^ conidia mg^− 1^, followed by Rich.-A, which produced 3.4 × 10^3^ conidia mg^− 1^. A lower number of conidia was retrieved from MEA (1.6 × 10^3^ conidia mg^− 1^).

**Fig. 3:**
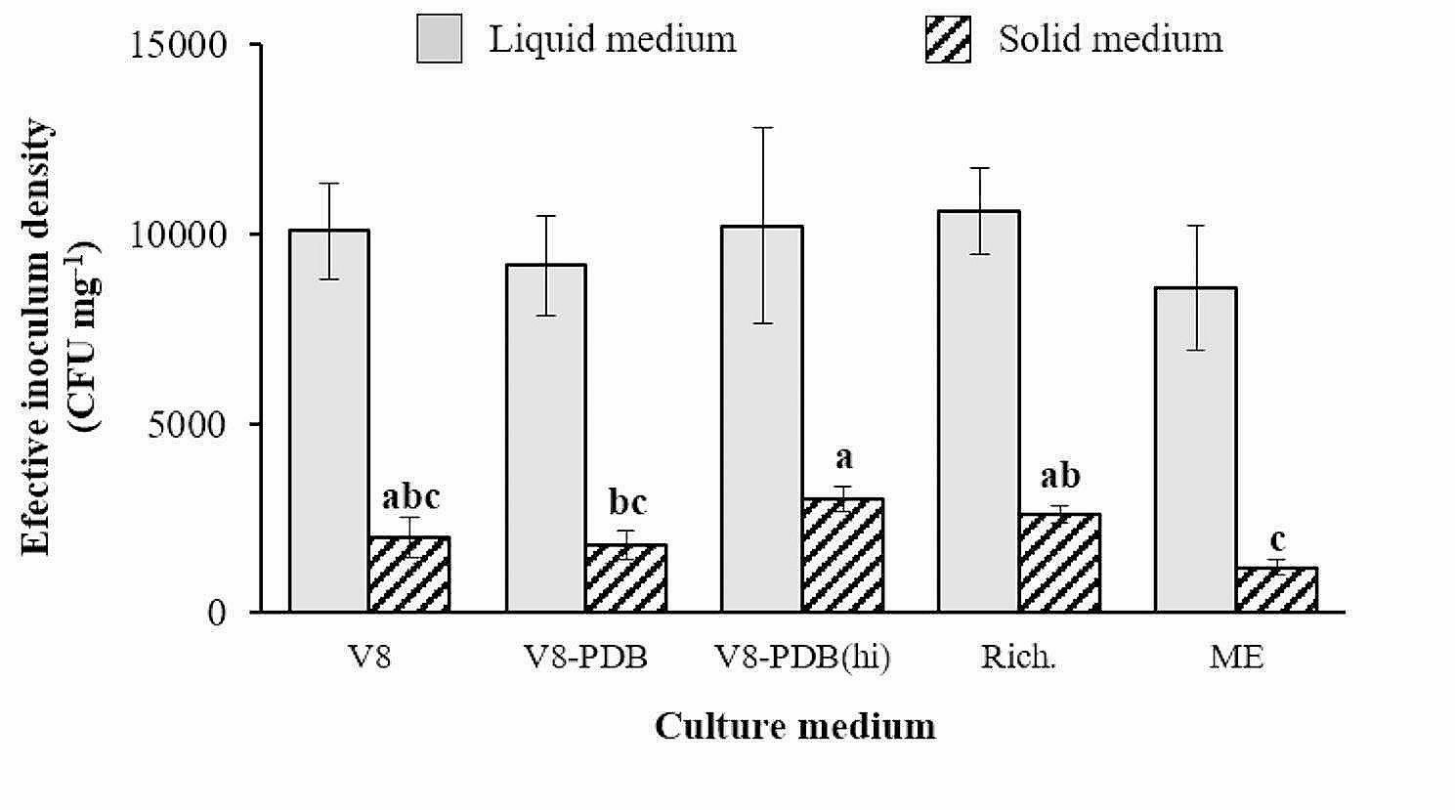
Comparison of the viability of the fungal material produced by the different axenic media. The graph shows the mean effective inoculum density expressed as CFU per mg of inoculum. Data presented are the mean values ± standard errors (vertical bars) for *n* = 3. Different minuscule letters indicate significant differences on solid media based on the LSD test (α ≤ 0.05)

Large significant differences in the number of colony forming units (CFU) were detected between liquid and solid media, with liquid formulations giving rise to approximately 25% more CFU mg^− 1^ than their solid counterparts (Fig. 3). Significant differences (*p* < 0.0001) were also detected between the viability of the fungal material produced by the solid media (Fig. 3), although the percentage of germinating spores was similar for all media, ranging from 72.8 (V8-PDA) to 82.1% (V8-A). In contrast, no statistically significant differences were detected between liquid media, all producing a similar number of CFU, ranging from 8.6 × 10^3^ (ME) to 1.1 × 10^4^ CFU mg^− 1^ (Rich.). Based on these findings, Richard’s medium was selected as the optimal medium for large-scale production of mycelial material and was used in subsequent experiments. Additionally, as the highest number of conidia was formed on V8-PDA(hi) medium, this medium was selected for mass production of conidia in subsequent experiments.

### Refinement of inoculum and screening conditions

Due to the challenge posed by the production of large quantities of *S. botryosum* conidia, which hinders large-scale screening, we assessed the infective capacity of its mycelium and tested different inoculum densities (1, 2.5 and 5 g mycelium L^− 1^ and 5 × 10^4^, 1 × 10^5^ and 2 × 10^5^ conidia mL^− 1^). Additionally, we examined the impact of RH on the progression of symptoms to improve and refine existing mass inoculation protocols.

Before inoculating plants with the different inoculum densities, we tested their viability and estimated their corresponding effective inoculum dose. An average of 1.5 × 10^4^ ± 2.5 × 10^3^, 4 × 10^4^ ± 3.5 × 10^3^ and 7.8 × 10^4^ ± 7.0 × 10^3^ CFU mL^− 1^ was obtained for the 1, 2.5 and 5 g L^− 1^ densities of ground mycelium and 9.8 × 10^3^ ± 2.6 × 10^3^, 3.2 × 10^4^ ± 6.6 × 10^3^ and 9.1 × 10^4^ ± 1.9 × 10^4^ CFU from the three conidia solutions (5 × 10^4^, 1 × 10^5^ and 2 × 10^5^ conidia mL^− 1^), respectively.

**Fig. 4:**
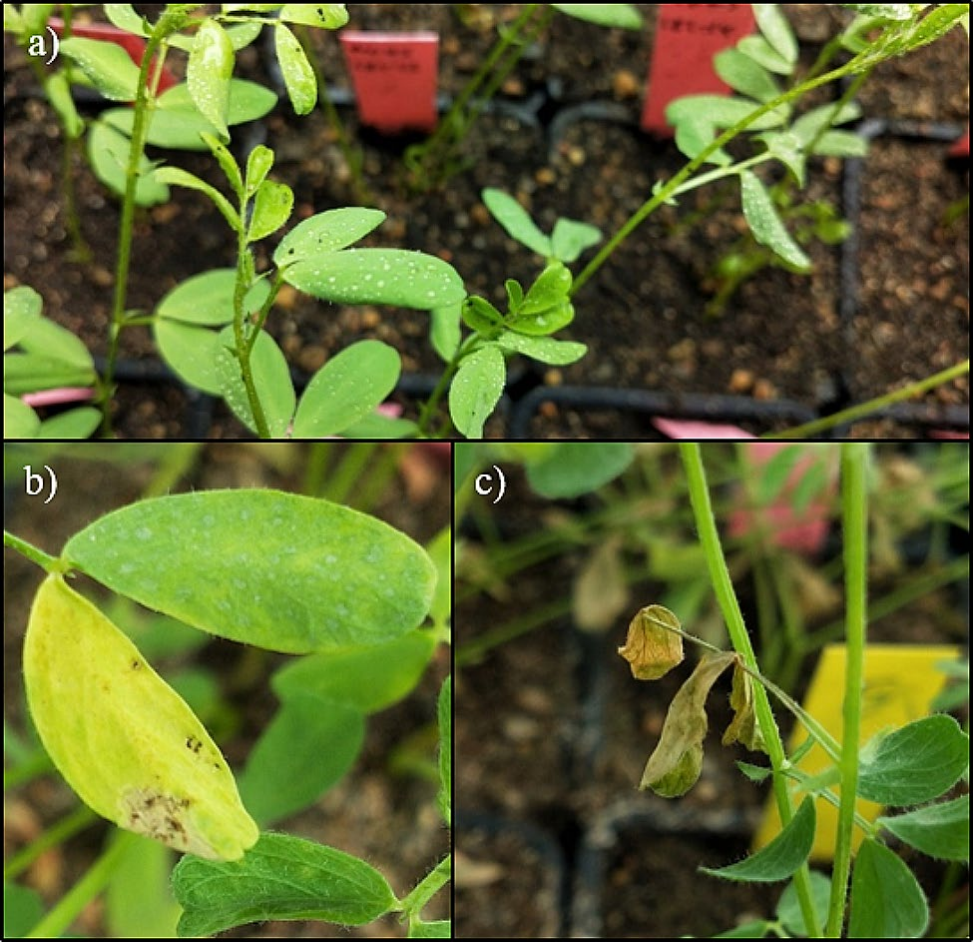
Plants responses to *Stemphylium botryosum* inoculation. (**a**) Initial plant condition post-inoculation, (**b**) development and progression of chlorotic lesions, and (**c**) complete wilting of leaves and early senescence

Stemphylium blight symptoms were observed on all genotypes, whereas no symptoms were observed on uninoculated plants. Mild symptoms were already evident after 48 h of incubation under high RH in heavily inoculated plants. By the end of the experiment (after 20 days), disease symptoms were noticeable on all inoculated plants (Fig. 4). The general ANOVA revealed significant differences between Genotype (G), Humidity (H), Inoculum Density (D) and the H$\times$D interaction for both Disease Rating and rAUDPC (Table S1).

**Fig. 5:**
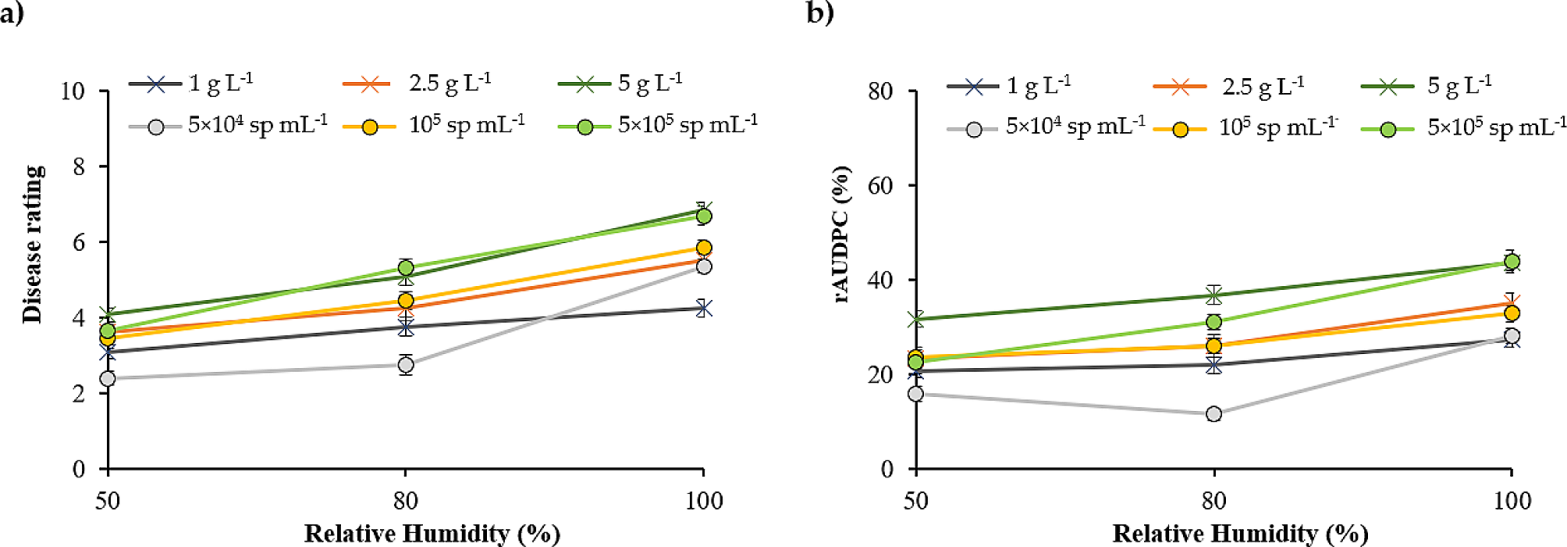
Relation between RH and disease rating (**a**) or rAUDPC (**b**) depending on the inoculum densities. Vertical bars are standard errors for *n* = 54

As shown in Fig. 5, disease severity ratings and rAUDPC increased with increasing levels of RH. Plants exposed to 100% RH showed a significantly higher mean rAUDPC than those exposed to 80% RH, while plants exposed to 50% RH exhibited the lowest symptom levels. To determine the specific effect of inoculum doses, data were analyzed separately for each RH level using a 2-way factorial design (Density×Genotype). The statistical values (F, *p*) are presented in Table S2. Significant differences were observed between inoculum densities under all RH conditions (50%, 80%, and 100%). In all cases, increasing the inoculum density led to higher levels of symptoms, observed both in terms of final disease severity ratings and rAUDPC.

To identify the optimal combination that clearly discriminates between genotypes, a randomized analysis of variance was performed for each combination of Humidity$\times$Density (Table S3). Although slight differences between genotypes were observed for almost all combinations, these differences were statistically significant for DR at 20 days post-inoculation (dpi) at the highest inoculum density and RH level (100%).

**Fig. 6:**
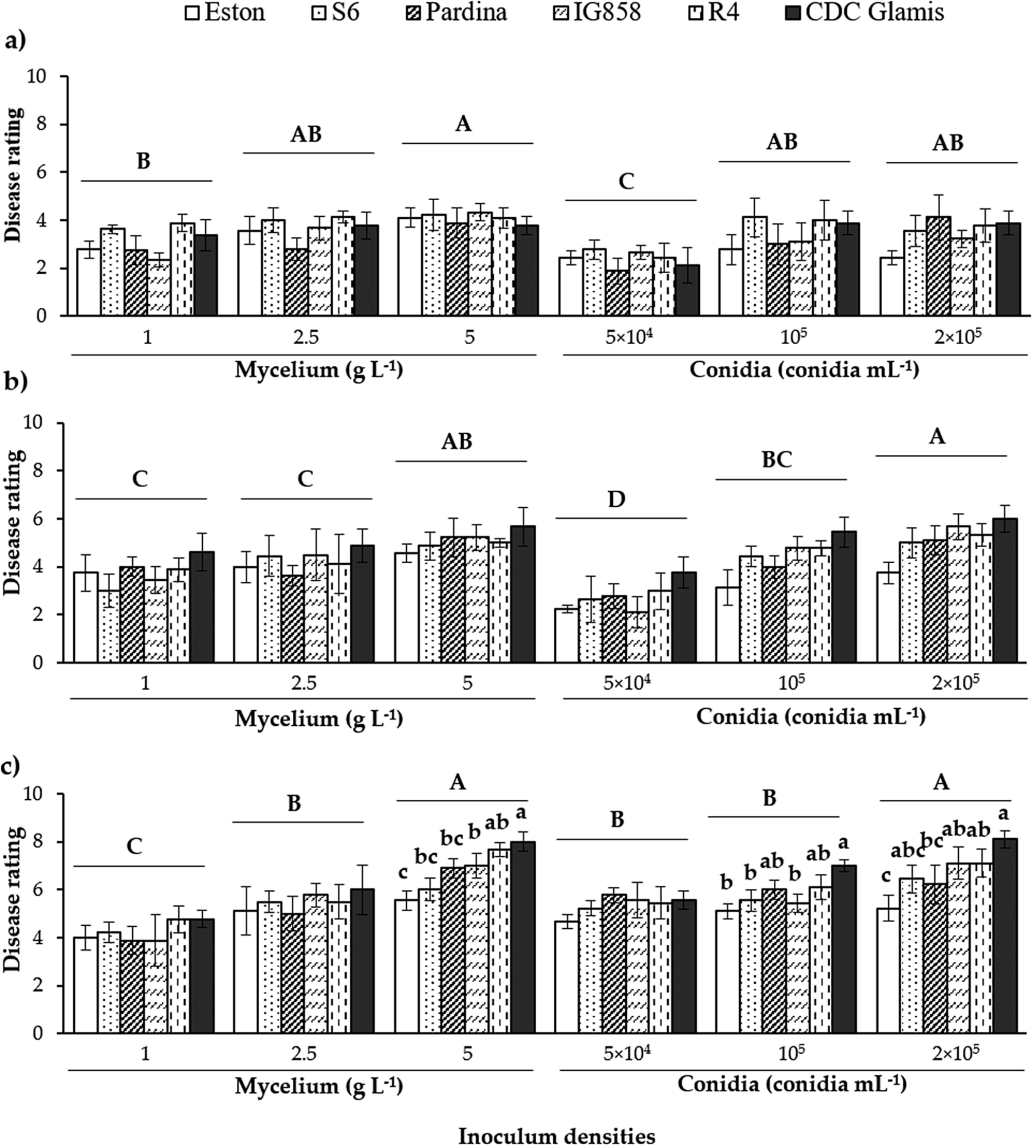
Average disease rating (DR) recorded on lentils inoculated with the isolate SB-2 of *Stemphylium botryosum* after 20 days. The results are presented based on air humidity conditions of (**a**) 50%, (**b**) 80%, and (**c**) 100% RH. Vertical bars are standard errors for *n* = 9. In each graph, densities marked with capital letters (**A**, **B**, **C**, or **D**) significantly differ according to the LSD test (α ≤ 0.05), and lowercase letters (**a**, **b**, or **c**) indicate significant differences between accessions for each Humidity$\times$Density combination, as determined by the LSD test (α ≤ 0.05)

Regarding DR (Fig. 5), the highest levels of symptoms were observed on plants inoculated with 5 g L^− 1^ mycelium or 2 × 10^5^ conidia mL^− 1^. At 50% and 80% RH, the recorded DR values were significantly lower (DR < 4 and DR < 5.8, respectively), and the differences detected between genotypes were not significant independently of the inoculum type and density (Fig. 6a, b). In contrast, at 100% RH (Fig. 5c), DR values were significantly higher. In addition, at this RH level, significant differences between genotypes could be observed after inoculation with the highest inoculum densities (5 g L^− 1^ of mycelium, 10^5^ conidia mL^− 1^ and 2 × 10^5^ conidia mL^− 1^) but not at the lowest densities. Under these combinations Humidity×Densities, the genotypes S6 and Pardina consistently appeared as the most resistant, displaying symptom levels similar to the resistant control (Eston). Genotype IG858 exhibited an intermediate level of symptoms, while genotypes R4 and CDC Glamis had the highest levels of symptoms.

**Fig. 7:**
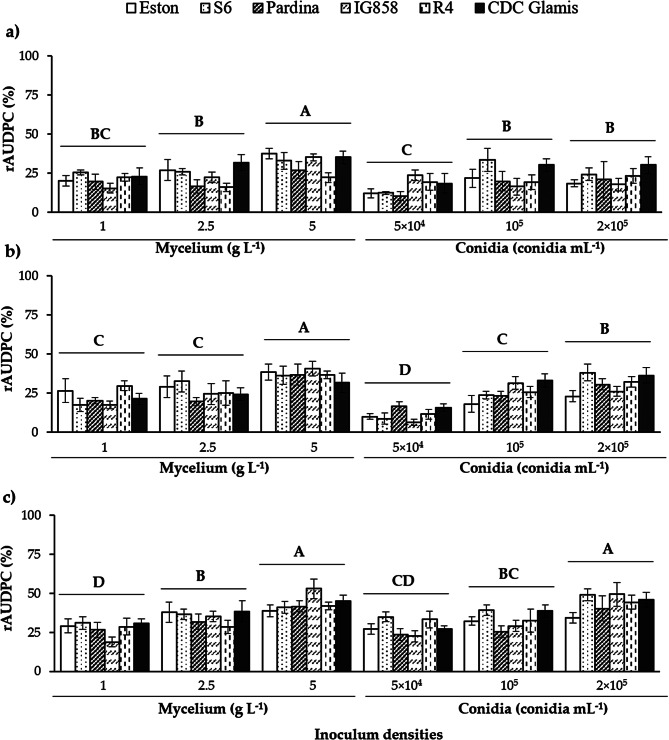
Relative Area Under the Disease Progression Curve (%) recorded on lentils inoculated with the isolate SB-2 of *Stemphylium botryosum* after 20 days. The results are based on air humidity conditions of (**a**) 50%, (**b**) 80% and (**c**) 100% RH. Vertical bars are standard errors for *n* = 9. For each graphic, densities with different capital letters (**A**, **B**, **C** or **D**) differ significantly according to the LSD test (α ≤ 0.05)

Consistent with the DR data (Fig. 7), an increase in inoculum density corresponded to higher rAUDPC values for both mycelium and conidia inoculum. At 100% RH (Fig. 6c), concentrations of 5 g mycelium L^− 1^ and 2 × 10^5^ conidia mL^− 1^ led to the highest rAUDPC values. At 50% and 80% RH (Fig. 6a, b), only the 5 g mycelium L^− 1^ inoculum resulted in significantly higher rAUDPC values. Unlike the DR results obtained, no significant differences were observed between genotypes at each Humidity x Density combination.

### Estimation of potential fungal inoculum for a mass scale inoculation

The results obtained from this study for the production of fungal material, alongside the refinement of inoculation methods and inoculum densities, are summarized in Table 1. The maximum number of plants that can be inoculated using either conidia (at the optimal density of 2 × 10^5^ conidia mL^− 1^) or mycelium (5 g mycelium L^− 1^) was estimated considering the requirement of 1 mL of inoculum for inoculation per plant.

**Table 1:** Summary of fungal material capacity for mass-scale inoculation for each culture medium. The table details the yield of fungal biomass, conidia densities, and the estimated number of plants that could be inoculated

Culture medium	Amount of fungal material (g L^− 1^ of media)		Amount of conidia (conidia L^− 1^)		Potential of inoculum (n° plants that can be inoculated L^− 1^ of medium)	
	Solid	Liquid	Solid	Liquid	Solid	Liquid
**V8**	0.5 ± 0.1	6.0 ± 1.0	2.4 × 10^6^	NP	5	1200
**V8-PDB**	0.6 ± 0.1	15.2 ± 1.6	2.5 × 10^6^	NP	7	3000
**V8-PDB(hi)**	0.6 ± 0.1	16.2 ± 0.8	3.8 × 10^6^	NP	11	3200
**Rich.**	0.6 ± 0.1	16.3 ± 0.8	3.4 × 10^6^	NP	10	3200
**ME**	0.6 ± 0.1	5.3 ± 0.7	1.6 × 10^6^	NP	4	1000

*Note * Spore production was not observed in liquid media (indicated as NP for ‘Not Produced’)

In a mass production scenario utilizing solid media, the average production of fungal material from a large tray (equivalent to 20 Petri dishes) ranged from 500 (V8-A) to 600 mg L^− 1^ (V8-PDA(hi)). This translates into a potential production of 1.6 × 10^6^ to 3.8 × 10^6^ conidia L^− 1^, enabling the inoculation of approximately 4 to 11 plants depending on the productivity of the medium.

In contrast, liquid medium formulations demonstrated a substantially higher yield of fungal material, with Richard’s medium producing 16.3 g L^− 1^. This amount is sufficient to prepare 3.2 L of inoculum at a concentration of 5 g L^− 1^, which could inoculate a corresponding number of 3200 plants, highlighting the efficiency of mycelium-based inoculation for mass screening purposes.

## Discussion

Reliable inoculum production is a primary requirement for research on plant breeding for pathogen resistance. This is particularly true for diseases for which scarce or very low levels of resistance have been detected thus far, such as stemphylium blight of lentil. In these cases, the evaluation of large germplasm collections is mandatory to broaden the resistance bases and increase the available level of resistance [23]. This requires the availability of suitable and efficient screening methods under controlled conditions ensuring (1) easy, rapid and abundant inoculum production, (2) fast and reproducible disease development, and (3) the capacity to discriminate between genotypes. Despite a few studies reporting the screening of moderately sized lentil germplasm collection against stemphylium blight [19, 21, 24], a protocol for lentil screening suitable for large-scale screening under laboratory conditions is not yet readily available. This is mainly due to the difficulty in producing high amount of viable conidia of *S. botryosum.* Refining the inoculation procedure and screening conditions to enable efficient and reproducible large-scale screening is therefore needed.

The consistent production of viable conidia by *S. botryosum* under laboratory conditions presents multiple challenges. The production of conidia in abundance is difficult, even when they are grown on PDA and/or V8 juice agar under alternate cycles of 12 h light and 12 h darkness [13]. Accordingly, we observed a very limited production of conidia in the different solid media tested, while growing the fungus in liquid media under constant agitation only led to vegetative growth (Fig. 2). The *S. botryosum* sporulation process is highly sensitive to environmental conditions, including humidity, light intensity, and temperature. Any variation in these parameters can significantly affect the quality and quantity of the conidia produced. The best media for *S. botryosum* conidia production were V8-PDA(hi) and Richard’s medium. However, even with the optimum medium and the use of large trays instead of Petri dishes, production is rather low, reaching 3.8 × 10^6^ L^− 1^ of medium (corresponding approx. to 20 Petri dishes). Despite these difficulties, different authors have reported artificial inoculation using conidial suspensions prepared from fungal axenic cultures to assess small-scale germplasm resistance under both controlled and semi controlled environments [8, 12, 22]. Accordingly, the use of large trays filled with V8-PDA(hi) medium allows the production of sufficient conidia to inoculate plants from medium-sized experiments (100–200 plants; Figs. 5 and 6). Nevertheless, the development of an efficient large-scale fungal material production technique has not been optimized to allow large pathogenicity studies due to the long time-consuming process and the diffident sporulating capabilities of *S. botryosum* in pure culture [11, 13].

Although recent investigations have turned to the use of mycelial suspensions for artificial inoculation [9], a thorough analysis of how the type and concentration of inoculum, along with growth conditions, interact with host genotypes remains incomplete. The data from our study suggest a significant advantage of mycelium as an inoculant for large-scale applications, particularly when compared to conidia-based methods using solid media. One liter of the most productive solid medium, V8-PDA(hi), can only produce enough inoculum to potentially infect a maximum of 11 plants at the optimal inoculum density, which is impractical for extensive screening experiments. Our results showed that mycelium-based inoculum was as virulent as conidia (Fig. 5). In addition, liquid medium provided a much higher quantity of viable fungal material since not only the quantity of fungal material collected from the media was 10 to 20 times higher in liquid than in solid media, but its viability was also 3 to 4 times higher (Figs. 1 and 3). This led to a marked increase in inoculum production when using liquid media, particularly Richard’s medium. Accordingly, 10 days of growth of *S. botryosum* in one liter of Richard’s medium is sufficient to generate 3.2 L of inoculum at a mycelial concentration of 5 g L^− 1^, allowing the inoculation of up to 3200 seedlings.

As stated earlier, environmental conditions, particularly temperature and moisture, are critical factors influencing the germination of conidia and subsequent disease development [12]. This requirement is not unique to *S. botryosum* in lentil; specific moisture conditions are similarly necessary for the onset of stemphylium leaf spot in alfalfa [25] and onion [26, 27], demonstrating the broad relevance of moisture in the disease cycle of the pathogen. Optimal conditions for disease proliferation have been identified as average day temperatures of 18–20 °C, high morning RH exceeding 85%, consistent rainfall, and the persistence of a thin moisture layer on leaf surfaces for over 48 h [7, 12, 18, 28]. The critical role of moisture in disease onset and development underscores the necessity of a controlled environment that can maintain these specific humidity and temperature parameters for successful infection and disease manifestation on lentil plants. Our study not only marks the infectivity of both conidia and mycelium but also determines the role of air humidity on disease progression. As expected, disease symptoms (yellowing, wilting and death of leaves and stems) were noticed on all infected plants [8]; however, significant genotypic differences became notably evident only at the highest inoculum densities and a RH of 100%. Although under field conditions the disease mainly occurs at an advanced growth stage, the period between 10 and 15 days post-germination is considered optimal for screening for various diseases [29, 30], facilitating the efficient use of resources and time. DR at 20 days post-inoculation offered a clear perspective on symptom severity; the most pronounced symptoms were associated with plants inoculated with 5 g L^− 1^ of mycelium, followed by the spore of 2 × 10^5^ conidia mL^− 1^. Contrastingly, at 50% and 80% RH, DR and their related rAUDPC were reduced, even at these inoculum densities, with no discernible genotypic differences at any inoculum concentration. In line with RH, we observed that increasing inoculum density was directly proportional to DR or rAUDPC, reaching the highest values for inoculum densities of 5 g L^− 1^ and 2 × 10^5^ conidia mL^− 1^. However, even under optimum conditions, this measurement did not allow to differentiate between genotypes.

## Conclusions

The data underscores the practicality of mycelium as a viable alternative to conidia-based inoculations for large-scale screenings. The high yield of mycelium in liquid media and its subsequent application as inoculum offers a streamlined pathway for resistance assessment, enabling the consistent and efficient inoculation of a substantial number of plants. According to these results, an improved inoculation method suitable for large-scale screening experiments is proposed. This method is based on the mass production of *S. botryosum* fungal material on Richard’s medium, a nutrient-rich substrate favorable for *S. botryosum* mycelial growth. It is recommended to grow the fungal isolate in this liquid medium for 10 days before collecting it by filtering under vacuum, drying it under sterile conditions and grinding the resulting dried fungal material to a fine powder. The inoculum can then be prepared by resuspending 5 g of ground fungal material into 1 L of sterile water. For the inoculation process, it is recommended to spray each lentil plant with 1 mL of this mycelial suspension and to incubate the inoculated plant for 48 h at 20 °C under 100% RH and complete darkness. After this initial period, optimum disease development will be achieved by maintaining the plants in a controlled environment under an RH close to 100%. These conditions are to be sustained over a period of 20 days, after which the foliar symptoms will be evaluated to measure the disease severity through the 0–10 Disease rating scale.

Given the minimal differences observed among genotypes and the substantial impact of RH and inoculum density, the reliability of less controlled screenings for inheritance studies could be considerably compromised. Unregulated environmental conditions could mask or amplify genetic resistance or susceptibility, leading to inconsistent or misleading results. Therefore, it is essential to conduct such screenings under well-defined and controlled conditions to ensure accurate and reproducible data that accurately reflect the genetic responses of the plants to the pathogen.

## Methods

### Plant material

A panel of six lentil genotypes was used in all experiments. The panel included one ICARDA selected line (IG 858), two breeding lines developed at IAS-CSIC derived from local selections within ICARDA germplasm (S6 and R4) [23] and a Spanish commercial brown type (Pardina). The cultivars Eston and CDC Glamis, which are partially resistant and susceptible to *S. botryosum*, respectively, were also used as positive and negative controls [19, 21]. Three plants of each genotype were grown in individual plastic pots (6 × 6 × 8 cm) containing 250 mL of a sand:peat mixture (1:1). Plants were maintained under controlled conditions (temperature of 25 ± 2 °C, 50% relative humidity (RH) and a photoperiod of 16/8 h day/night regime) and watered when needed with tap water.

### Fungal material

One isolate of *S. botryosum* (SB2), which was originally obtained from stemphylium blight lesions on lentils (kindly provided by the Pulse Crop Pathology Research Group of the Crop Development Centre, University of Saskatchewan, Canada), was used across all experiments. The fungal isolate was selected on the basis of its high aggressiveness, as determined in a previous screening performed under controlled conditions comparing four different isolates (unpublished data). Before starting the experiments, the *S. botryosum* isolate was first refreshed on V8-PDA Petri dishes at a temperature of 25 ± 2 °C under a 16 h photoperiod for 10 days. Subsequently, the isolate was purified through single-spore isolation and maintained on V8-PDA plates at the same temperature and photoperiod conditions for 10 days.

### Optimization of *Stemphylium botryosum* conidia and mycelium production

To determine the optimum growth medium for conidia and mycelium formation, the fungal material from three 10-day-old *S. botryosum* culture plates on V8-PDA was collected by scraping the medium surface and resuspended in 100 mL of sterile water. One mL of this suspension was then inoculated on five replicated 10 × 10 cm agar plates (for solid media) and 250 mL flasks (for liquid media) of Malt extract (ME: 20 g L^− 1^ malt extract, 1 g L^− 1^ peptone, 20 g L^− 1^ sucrose), V8 juice agar (V8A: 200 mL L^− 1^ of Campbell’s V8 juice, 4 g L^− 1^ CaCO_3_), V8 + PDB (V8-PDB: 150 mL L^− 1^ V8 juice, 10 g L^− 1^ PDB), V8 + PDB high (V8-PDB(hi): 250 mL L^− 1^ V8 juice, 5 g L^− 1^ CaCO_3_, 15 g L^− 1^ PDB, 2.5 g L^− 1^ MgSO_4_·7 H_2_O, 15 mg L^− 1^ FeCl_3_) and modified Richard’s medium (Rich.: 10 g L^− 1^ sucrose, 10 g L^− 1^ KNO_3_, 5 g L^− 1^ KH_2_PO_4_, 2.5 g L^− 1^ MgSO_4_·7 H_2_O, 20 mg L^− 1^ FeCl_3_, 150 ml L^− 1^ V8 juice, pH = 6.0). For solid medium, each formulation was solidified by adding 13 g L^− 1^ agar (V8-PDA and V8-PDA(hi)) or 15 g L^− 1^ (MEA, V8A and Rich.-A). Inoculated plates were incubated at 25 ± 2 °C under a 16 h photoperiod for 25 days before air-drying under sterile conditions. The fungal material was then collected by scraping the dried medium surface. Meanwhile, the flasks were incubated at 25 °C in an orbital shaker (Inforst-HT Ecotron, Bottmingen-Basel, Switzerland) at 110 rpm. After 10 days of growth, the fungal material was vacuum-filtered, dried under sterile conditions, and ground into a fine powder with a grinder. After complete desiccation, the fungal material recovered from each plate and flask was weighed. To quantify the conidia produced from each plate and flask, 10 mg of dried fungal material was added to 20 mL Falcon tubes containing 10 mL of sterile water and quantified with a haemocytometer (Fuchs-Rosenthal chamber). In parallel, the fungal material produced from five replicates of each medium was combined and homogenized. Ten milligrams of each mixture were then added to 20 mL Falcon tubes containing 10 mL of sterile water to determine their viability.

### Mass preparation of conidial and mycelial material

For large-scale conidia production, the fungal material from two 10 days-old *S. botryosum* (SB2) culture plates on V8-PDA was collected by scraping the medium surface and resuspended in 20 mL of sterile water. The fungal suspension was evenly spread onto a sterile baking tray (25 × 50 × 3 cm) containing 700 mL of V8-PDA (hi) medium. After sealing the tray with sterile cling film, it was incubated at 25 °C under a 16 h photoperiod for 25 days. The medium was then air-dried under sterile conditions, and the dried medium surface was scraped to collect the conidia. To ensure complete dryness, the collected spores were further dried in a beaker at 30 °C for 24 h and subsequently stored at 4 °C.

For large-scale mycelium production, 20 mL of fungal suspension obtained as previously described was transferred into 500 mL of Rich. and incubated at 25 °C in an orbital shaker (Inforst-HT Ecotron, Bottmingen-Basel, Switzerland) at 110 rpm. After 10 days of growth, the mycelium was vacuum-filtered, dried under sterile conditions, ground into a fine powder and stored at 4 °C.

### Viability test and estimation of effective inoculum density

To assess the viability of the different inocula tested and of the fungal material produced by each medium, each inocula and fungal material solution was diluted at a 1:100 ratio, homogeneously plated on three plates of PDA (100 µL plate^− 1^), and incubated for two days at 25 °C. Individual *S. botryosum* colonies were then counted, and the effective inoculum density was estimated as colony forming units (CFU) per mL.

### Optimization of inoculation procedure

To determine the optimum inoculum type, dose, and humidity condition for disease development, a trial was conducted on lentil plants using *S. botryosum* conidia or mycelium. Three different mycelium solutions (1, 2.5 and 5 g of ground mycelium L^− 1^) and three different conidia solutions (5 × 10^4^, 1 × 10^5^ and 2 × 10^5^ conidia mL^− 1^) were prepared and tested independently. The fungal material (conidia or mycelium) was added to sterile water containing 0.01% agar and 0.03% Tween^®^ 20 (v:v) as a wetting agent. Nine three to five leaf stage seedlings (10–15 days old) were inoculated per lentil genotype, inoculum type, and inoculum density. Additionally, nine plants per lentil genotype were used as uninoculated negative controls. After inoculation, lentils were incubated for 48 h in complete darkness and high RH conditions (100% RH). To determine the optimum RH for disease development, three plants per lentil genotype, inoculum type and inoculum density were transferred to growth chambers, with temperature and photoperiod conditions as previously described, with varying RH conditions as follows: (i) a growth chamber without additional humidity (40% RH); (ii) a growth chamber including two additional humidifiers (Saivod CF-2880 Ultrasonic Humidifiers, Madrid, Spain), operating at a rate of 10 min per hour (80% RH); and (iii) a growth chamber were the plants, along with the humidifiers operating as in point ii), were covered with a transparent plastic film (100% RH). Inoculated and uninoculated plant genotypes were kept separately to prevent fungal cross-contaminations. Foliar symptoms were assessed using a semi-quantitative disease rating (DR) scale, measuring plant damage as follows: 0 = healthy plant; 1 = 1 to 10% of plant surface infected with tiny lesions; 2 = 10 to 20% of plant damaged with a few chlorotic lesions; 3 = 21 to 30% of plant with expanding lesions on leaves and beginning of leaf drop); 4 = 31 to 40% of plant (approximately 1/5 of nodes involved) showing symptoms and leaf drop; 5 = 41 to 50% of plant (approx. 2/5 of nodes affected) with lesions; 6 = 51 to 60% of plant (approx. 3/5 of nodes affected) with symptoms; 7 = 61 to 70% of plant with symptoms (4/5 of nodes affected); 8 = 71 to 80% of plant affected (all leaves dried); 9 = 81 to 90% of plant affected (all leaves dried up, and stem started to dry); and 10 = 91 to 100% of diseased plant (completely dead) [19, 22]. Symptoms were measured at 0, 2, 5, 10, 15 and 20 days after inoculation. Periodic assessments of disease severity were then used to estimate the rAUDPC parameter [31].

### Statistical analysis

To determine the optimum medium for the production of *S. botryosum* conidia and mycelium, a randomized design was employed. To assess differences among inoculum type, density and humidity conditions, a split-split-plot design with three blocks was used. Each block was composed of three plants, and it featured eight levels of inoculum as the main plot, three RH levels as the subplot, and six lentil genotypes as the subplot.

Whenever the ANOVA test yielded significant results at *p* < 0.05, a mean comparison test was performed by LSD test at α = 0.05. All statistical analyses were carried out using Statistix 9.0 software (Tallahassee, FL, USA).

### Electronic supplementary material

Below is the link to the electronic supplementary material.

Supplementary Material 1

## Data Availability

No datasets were generated or analysed during the current study.
