# Supplementary material for: Optimization of inoculum production of Stemphylium botryosum for large-scale resistance screening of lentils
Source: Plant Methods. 2024 Apr 4;20:51. doi: 10.1186/s13007-024-01177-4 (PMC10993456; doi:10.1186/s13007-024-01177-4)
Supplement: Supplementary file 1 — Supplementary Material 1 [file 13007_2024_1177_MOESM1_ESM.docx]

**Table S1.** Statistical values (F and *p*) and degrees of freedom (*df*) of the general factorial analysis of final DR and rAUDPC (%) for the variables: relative humidity (H), inoculum density (D), genotype (G) and their interactions.

|  | **Humidity (H)** | **Density (D)** | **Genotype (G)** | **H×D** | **H×G** | **D×G** | **H×D×G** |
| --- | --- | --- | --- | --- | --- | --- | --- |
| Disease rating | **F=151.34; *p*<0.0001** | **F=29.98; *p*<0.0001** | **F=8.75; *p*<0.0001** | **F=3.64; *p*=0.0001** | F=1.49; *p*=0.1379 | F=0.81; *p*=0.7295 | F=0.38; *p*=1.0000 |
| rAUDPC (%) | **F=73.65; *p*<0.0001** | **F=38.51; *p*<0.0001** | **F=4.74; *p*=0.0003** | **F=3.05; *p*=0.0008** | F=0.76; *p=*0.6678 | **F=1.66; *p*=0.0229** | F=0.86; *p*=0.7384 |
| *df* | 2 | 5 | 5 | 10 | 10 | 25 | 50 |

|  | **Humidity** | **Density (D)** | **Genotype (G)** | **D×G** |
| --- | --- | --- | --- | --- |
| Disease rating | 50% | **F=6.79; *p*<0.0001** | **F=2.43; *p*=0.0354** | F=0.58; *p*=0.9495 |
|  | 80% | **F=12.48; *p*<0.0001** | **F=3.27; *p*=0.0070** | F=0.44; *p*=0.9911 |
|  | 100% | **F=17.47; *p*<0.0001** | **F=6.13; *p*<0.0001** | F=0.58; *p*=0.9493 |
| rAUDPC (%) | 50% | **F=7.46; *p*<0.0001** | **F=3.36; *p*=0.0058** | F=1.08; *p*=0.3708 |
|  | 80% | **F=21.96; *p*<0.0001** | F=0.52; *p*=0.7630 | F=1.25; *p*=0.1930 |
|  | 100% | **F=15.46; *p*<0.0001** | **F=2.26; *p*=0.0483** | F=1.05; *p*=0.4002 |
| *df* |  | 5 | 5 | 25 |

**Table S2**. Statistical values (F and *p*) and degrees of freedom (*df*) of the two-way factorial analysis of final disease rating and rAUDPC (%) between the inoculum doses (D), genotype (G) and the interactions between them per humidity condition.

**Table S3.** Statistical values (F and *p*) of a randomized analysis of variance of final disease rating and rAUDPC (%) per humidity condition and inoculum dose (*df* = 5). Values in bold reflect significant differences among accessions.

| **Humidity** | **Symptoms** | **Mycelium (g L^-1^)** | | | **Conidia (conidia mL^-1^)** | | |
| --- | --- | --- | --- | --- | --- | --- | --- |
|  |  | **1** | **2.5** | **5** | **5×10^4^** | **10^5^** | **2×10^5^** |
| **50%** | Disease rating | F=1.87; *p=*0.1188 | F=0.89; *p* =0.4946 | F=0.17; *p* =0.9707 | F=0.47; *p* =0.7978 | F=0.65; *p* =0.6657 | F=1.44; *p* =0.2277 |
|  | rAUDPC (%) | F=0.89; *p* =0.4942 | F=2.33; *p* =0.0579 | F=2.09; *p* =0.0828 | F=1.95; *p* =0.1051 | F=1.47; *p* =0.2187 | F=0.67; *p* =0.6449 |
| **80%** | Disease rating | F=0.77; *p* =0.5770 | F=0.25; *p* =0.9358 | F=0.41; *p* =0.8420 | F=0.83; *p* =0.5339 | F=2.23; *p* =0.0668 | F=2.04; *p* =0.0904 |
|  | rAUDPC (%) | F=0.89; *p* =0.4942 | F=0.52; *p* =0.7571 | F=0.33; *p* =0.8936 | F=2.30; *p* =0.0603 | F=2.05; *p* =0.0891 | F=2.02; *p*=0.0938 |
| **100%** | Disease rating | F=0.43; *p* =0.8279 | F=0.37; *p* =0.8686 | **F=4.96; *p* =0.0002** | F=0.64; *p* =0.6682 | **F=2.88; *p* =0.0236** | **F=2.66; *p* =0.0334** |
|  | rAUDPC (%) | F=1.28; *p* =0.2876 | F=0.79; *p* =0.5633 | F=1.56; *p* =0.1883 | F=1.85; *p* =0.1217 | F=1.54; *p* =0.1966 | F=1.05; *p* =0.3998 |
